# Axial strength of back to back cold formed steel short channel sections with unstiffened and stiffened web holes

**DOI:** 10.1038/s41598-025-15992-9

**Published:** 2025-08-21

**Authors:** Ardalan B. Hussein, Ferenc Papp

**Affiliations:** Department of Structural Engineering and Geotechnics, Széchenyi István University, Egyetem Tér 1, Gyor, 9026 Hungary

**Keywords:** Axial load capacity, Built-up CFS columns, Cold-formed steel (CFS), Direct strength method (DSM), Edge-Stiffened holes, Nonlinear finite element model, I-section columns, Unperforated webs, Unstiffened holes, Web hole, Web opening, Web perforation., Engineering, Civil engineering

## Abstract

The increasing adoption of back-to-back built-up cold-formed steel (CFS) channel columns in construction is attributed to their lightweight nature, versatility in shape fabrication, ease of transportation, cost efficiency, and enhanced load-bearing capacity. Additionally, the incorporation of web openings facilitates the integration of electrical, plumbing, and heating systems. These built-up sections are widely utilized in wall studs, truss elements, and floor joists, with intermediate screw fasteners strategically positioned at regular intervals to prevent the independent buckling of channels. Based on 18 experimental tests, this study demonstrates an excellent correlation between finite element analysis and the experimental results, confirming the accuracy of geometrically and materially nonlinear finite element modeling in predicting the axial buckling strength of built-up short columns. Furthermore, the design standards of the American Iron and Steel Institute and Australian/New Zealand Standards were found to underestimate the axial load capacity by approximately 12.5%. The primary objective of this research is to investigate the influence of various hole configurations, both with and without stiffeners, on the axial performance of built-up short CFS channel columns. A total of 180 finite element models were developed, examining four different unstiffened and edge-stiffened hole configurations, validated against experimental results from plain webs. The findings reveal that web holes and edge stiffeners significantly impact axial load-bearing capacity, while the specific shape of the openings has a negligible effect. Specifically, introducing a hole at the centroid of each web results in an approximate 8.5% reduction in axial load capacity in the absence of edge stiffening. However, the incorporation of stiffeners around the perforations mitigates this reduction and enhances both structural efficiency and load-bearing capacity. These results highlight the critical role of edge stiffening in optimizing the structural performance of perforated built-up CFS columns.

**Supplementary Information:**

The online version contains supplementary material available at 10.1038/s41598-025-15992-9.

## Introduction

Cold-formed steel (CFS) sections have become increasingly popular in structural applications due to their numerous advantages over traditional materials such as concrete and timber. These benefits include high strength-to-weight ratio, recyclability, dimensional accuracy, and ease of prefabrication, handling, transportation, and installation. Additionally, CFS members do not experience shrinkage or creep at room temperature, making them a reliable choice for modern construction systems^1–3^. The primary manufacturing processes for CFS members involve cold roll forming, and press^4^ and bending^2^ braking operations. Among various built-up CFS configurations, the I-section, formed by connecting back-to-back lipped channel sections using screws, welds, or bolts, is widely adopted due to its symmetrical geometry and efficient load-carrying capacity^5^.

Cold-formed steel (CFS) sections are produced by shaping thin steel sheets at or near room temperature using methods such as bending brakes, press brakes, or roll-forming machines. In contrast, hot-rolled steel sections are manufactured by rolling steel at elevated temperatures above the recrystallization point. CFS members typically have thin walls, which makes them more susceptible to local buckling, whereas hot-rolled sections, being thicker and more rigid, provide greater resistance to such instability^6^.

The structural performance of built-up CFS columns is highly dependent on several factors, including the spacing and arrangement of fasteners. Studies have shown that intermediate spacing influences the failure mechanisms of CFS built-up sections^7^. In particular, the distribution of screws^8^ and the end fastener group (EFG)^5^ significantly affect the axial strength, especially in columns that exhibit global buckling^9^. While some researchers argue that reducing fastener spacing or introducing EFG does not enhance the local-distortional strength of built-up I-section columns^10^, others have demonstrated that increasing the number of screws has a minimal effect on stub columns but a considerable impact on the strength of short and intermediate columns when screw spacing is doubled^11^. Moreover, several other studies^12–14^ have also focused on the behavior of built-up CFS sections. In addition, some research has been conducted on perforated high-strength stainless steel girders^15–18^.

Perforations are often introduced into CFS columns to accommodate utility access, reduce weight, or facilitate construction practices. However, the presence of web openings alters the stress distribution and affects axial strength, particularly in compression members. Approximate strength assessments suggest that the effect of holes on axial and flexural members can be neglected if the cumulative hole length does not exceed 10% of the total member length, the maximum hole depth is at least 25% of its length, and the net cross-sectional area is at least 95% of the gross area^6^. Members that meet these criteria generally experience a capacity reduction of 5% or less due to web openings. However, the shape, size, and distribution of holes can significantly influence failure behavior.

Given the advantages and widespread application of CFS sections^19–26^, several studies have explored the effects of web perforations on the axial capacity of CFS members. Chen et al^27,28^ found that unstiffened circular holes negatively impact built-up I-section columns, whereas edge-stiffened circular holes can slightly enhance their strength. He et al^29^ further observed that web holes significantly affect the elastic stiffness of stub columns but have a negligible impact on slender columns. Moreover, elevated temperatures drastically reduce the strength of both plain and perforated back-to-back I-channels, with axial capacity decreasing by approximately 85% as temperature rises from 20 to 700 °C^30^. The influence of perforations is particularly noticeable in short columns, where hole dimensions and configurations alter performance, whereas intermediate and long columns exhibit relatively unaffected ultimate loads^31^.

The specific geometry of web perforations also plays a crucial role in determining structural performance. Zhao et al^32^ found that slotted web holes shift the location of local buckling from the maximum initial imperfection zone to the perforation region, reducing axial capacity, while He et al^33^ observed that slotted perforations have only a marginal negative effect on stiffness and load-bearing capacity. Similarly, Chandramohan et al^34^ demonstrated that elongated unstiffened web holes and elongated edge-stiffened web holes lead to approximately 15% and 3% reductions in axial strength, respectively. In contrast, some studies suggest that certain hole configurations have a negligible effect. For instance, Aktepe et al^35^ reported that circular holes exert a minor influence on failure behavior and ultimate strength, and intermediate columns with and without perforations exhibit similar sensitivities to geometric imperfections. Meanwhile, Chen et al^36^ found that increasing the stiffener length and fillet size in edge-stiffened circular holes slightly improves axial strength. However, as the diameter of circular holes increases, the ultimate load capacity of Carbon Fiber Reinforced Polymer (CFRP)-strengthened columns decreases^37^. The presence of web perforations also alters failure modes, with CFRP-reinforced specimens exhibiting local-distortional interactive deformations at the perforation site^38^. Furthermore, research by Kulatunga et al^39^ indicates that current American Iron and Steel Institute (AISI) specifications tend to underestimate the axial capacity of perforated CFS columns.

Despite the extensive research on the influence of web perforations on CFS members, limited studies have systematically analyzed the axial load behavior of built-up CFS I-section short columns with different web hole configurations. Therefore, this study aims to numerically investigate the impact of unstiffened and edge-stiffened circular, rectangular, square, and slotted web openings on the axial strength of built-up back-to-back cold-formed steel channel short columns. A validated finite element model (FEM) is employed to assess the effects of these perforations on structural performance, providing valuable insights for optimizing the design and load-bearing capacity of perforated CFS columns.

## Design of compression members

**Fig. 1:**
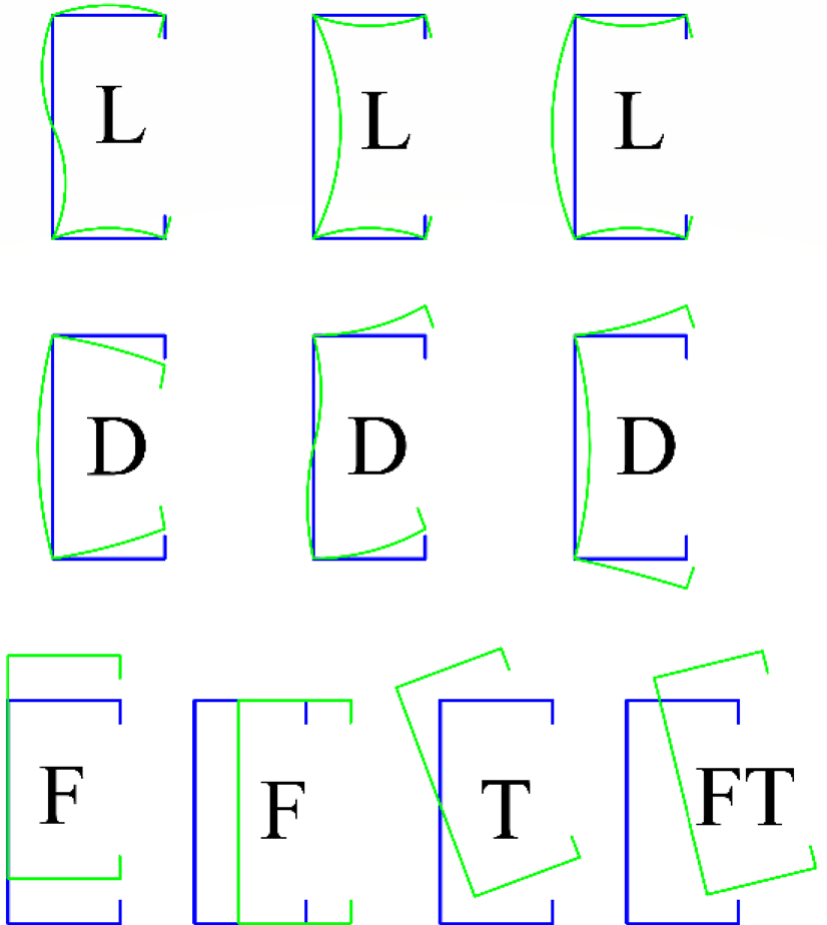
Types of buckling in cold-formed steel columns ^41^.

Local buckling (L) refers a buckling limit state in which a compressed plate element deforms independently, while the intersection lines between connected elements remain straight and the angles between them are preserved. In contrast, distortional buckling (D) involves a change in the cross-sectional geometry but does not include local buckling^6,40^. Global buckling (G) occurs without any distortion of the cross-section, while flexural buckling (F) is characterized by a compression member deflecting laterally without any twisting or change in cross-sectional shape. Torsional buckling (T) involves a compression member twisting about its shear center axis, and flexural–torsional buckling (FT) a buckling mode in which a compression member undergoes simultaneous bending and twisting, while the cross-sectional shape remains unchanged^6^, as shown in Fig. 1.

The Direct Strength Method (DSM) is a design technique that predicts the resistance of CFS members directly, without relying on effective dimensions, unlike the Effective Width Method (EWM). The EWM accounts for local deformation by reducing the total cross-sectional area under a non-linear stress distribution to an effective area under a simplified linear stress distribution^6^.

Compared to the EWM, the DSM is generally considered the more modern, accurate, and efficient approach for designing Cold-Formed Steel (CFS) sections. According to AS/NZS^40^, the axial load capacity of CFS sections is determined using DSM, as specified in the following equations:

### General

The nominal capacity of a compression member (N_c_) should be determined as the lesser of the nominal capacities for local buckling (N_cl_), distortional buckling (N_cd_), and global buckling (N_ce_). In contrast, P_AS/NZS_ is used in Table 2 as a substitute for N_c_.

### Global buckling (flexural, torsional or flexural–torsional buckling)

The nominal yield capacity of a compression member is represented by N_y_. For buckling behavior, the minimum elastic compression member buckling load for global buckling is denoted as N_oc_, while the nominal capacity of a member under compression concerning global buckling is denoted as N_ce_. Global buckling is further categorized into flexural (F), torsional (T), or flexural–torsional (FT) buckling, as illustrated in Fig. 1. Additionally, f_oc_ represents the elastic flexural buckling stress, λ_c_ represents the column global slenderness, and A_g_ denotes the gross area of the cross-section.

1 $${\text{N}}_{{{\text{oc}}}} = {\text{ Ag }} \times {\text{f}}_{{{\text{oc}}}}$$

2 $${\text{N}}_{{\text{y}}} = {\text{ Ag }} \times {\text{ f}}_{{\text{y}}}$$

3 $$\lambda_{{\text{c}}} = \sqrt {\frac{{{\text{N}}_{{\text{y}}} }}{{{\text{N}}_{{\text{oc }}} }}}$$

4 $${\text{If}}\,\, \lambda_{c} \le 1.5 ,\,\,{\text{N}}_{ce} = {\text{N}}_{y} \left( {0.658^{{\lambda_{c}^{2} }} } \right)$$

5 $${\text{If}}\,\, \lambda_{c} > 1.5,\,\,{\text{N}}_{ce} = {\text{N}}_{y} \left( {\frac{0.877}{{\lambda_{c}^{2} }}} \right)$$

### Local buckling

The nominal member capacity in compression is denoted as N_cl_, while N_ol_ represents the elastic local buckling load, f_ol_ signifies the elastic local buckling stress, and ${\uplambda }_{{\ell}}$ represents the column local slenderness.

6 $${\text{N}}_{{{\text{ol}}}} = {\text{ f}}_{{{\text{ol}}}} \times {\text{Ag}}$$

7 $${\uplambda }_{{\ell}}=\sqrt{{\text{N}}_{\text{ce}}/{\text{N}}_{\text{ol}}}$$

8 $${\text{If}}\,\, \lambda_{l} > 0.776,\,\, {\text{N}}_{cl} = \left( {\frac{{N_{ol} }}{{N_{ce} }}} \right)^{0.4} \,\,{\text{N}}_{ce} \left[ {1 - 0.15\left( {\frac{{N_{ol} }}{{N_{ce} }}} \right)^{0.4} } \right]$$

9 $${\text{If }}\,\,{\uplambda }_{{\text{l}}} \le 0.776,\,\,{\text{ N}}_{{{\text{cl}}}} = {\text{N}}_{{{\text{ce}}}}$$

### Distortional buckling

The nominal member capacity for a compression member in relation to distortional buckling is denoted as N_cd_. The elastic distortional compression member buckling load is represented by N_od_, the elastic distortional buckling stress is written as f_od_, and λ_d_ represents the column distortional slenderness.

10 $$N_{od} = {\text{ f}}_{od} \times {\text{A}}_{g}$$

11 $$\lambda_{d} = \sqrt {\frac{{N_{y} }}{{N_{od} }}}$$

12 $$I{\text{f}}\,\, \lambda_{d} > 0.561,\,\, {\text{N}}_{cd} = {\text{N}}_{y} \left( {\frac{{N_{od} }}{{N_{y} }}} \right)^{0.6} \left[ {1 - 0.25\left( {\frac{{N_{od} }}{{N_{y} }}} \right)^{0.6} } \right]$$

13 $${\text{If}}\,\,\lambda_{ d} \le 0.561,\,\,{\text{N}}_{{{\text{cd}}}} = {\text{N}}_{{{\text{y}}}}$$

14 $${\text{N}}_{{\text{c}}} = {\text{ Minimum}}\,\,{\text{ of}}\,\, \, \left( {{\text{N}}_{{{\text{cd}}}} ,\,{\text{N}}_{{{\text{cl}}}} ,\,{\text{and}}\,{\text{N}}_{{{\text{ce}}}} } \right)$$

### Cross-section imperfections

Advanced analysis should consider cross-section imperfections, including those related to local and distortional buckling, as outlined below:

1. Imperfections in the shapes of local and distortional buckling modes must be incorporated into the structural model. This is achieved by applying imperfection multipliers to the local and distortional buckling modes, which assume unit maximum deformation, and superimposing these scaled imperfections onto the ideal geometry.
2. The imperfection multipliers for local buckling (S_ol_) will be determined in accordance with the following:
  15 $${\text{S}}_{{{\text{ol}}}} = \, 0.{3}\, \times \,{\text{t}}\, \times \,\sqrt {\frac{{{\text{f}}_{{\text{y}}} }}{{{\text{f}}_{{{\text{ol}}}} }}}$$
3. The imperfection multipliers for distortional buckling (Sod) will be calculated using the following formula:
  16 $${\text{S}}_{{{\text{od}}}} = \, 0.{3}\, \times \,{\text{t}}\, \times \,\sqrt {\frac{{{\text{f}}_{{\text{y}}} }}{{{\text{f}}_{{{\text{od}}}} }}}$$

In these equations, t represents the thickness of the plate, f_ol_ denotes the elastic local buckling stress, and f_od_ refers to the elastic distortional buckling stress.

## Experimental tests were used for validation of the finite element model

### Specimen preparation

The columns were fabricated from galvanized CFS, with a nominal thickness of 1.2 mm. Prior to assembling the built-up I-section columns, the precise cross-sectional dimensions of each specimen were carefully measured. The average values of these measurements are provided in Table 1.

**Table 1:** Measured dimensions and screw spacing of tested specimens ^42^.

Specimen	Left Section						Right Section						Length	Screw Spacing	
	Web Depth	Flange Length		Lip Length		Thickness	Web Depth	Flange Length		Lip Length		Thickness			
	D	B1	B2	d1	d2	t	D	B1	B2	d1	d2	t	L	S1	S
BU45-7-1	122	55	54	23	15	1.18	123	54.5	53.5	23	15	1.18	359	45	45
BU45-7-2	121	53	52	22	14	1.17	123	55	54	23	15	1.18	366	45	45
BU45-7-3	122	54	53	22.5	14.5	1.17	122	53	52	22	14	1.17	360	45	45
BU90-3-1	121	53	52	22	14	1.18	122	54	53	22.5	14.5	1.18	359	90	90
BU90-3-2	123	53.5	52.5	22.5	14.5	1.17	124	55	54	23	15	1.17	361	90	90
BU90-3-3	122	53.5	52.5	22.5	14.5	1.18	122	53.5	52.5	22.5	14.5	1.17	359	90	90
BU105-2-1	121	52	51	21.5	13.5	1.17	122	54	53	22.5	14.5	1.18	358	105	150
BU105-2-2	122	54	53	22.5	14.5	1.16	124	56	55	23.5	15.5	1.17	365	105	150
BU105-2-3	121	54	53	22.5	14.5	1.18	122	54	53	22.5	14.5	1.17	363	105	150
BU60-7-1	141.5	51.5	52.5	23.5	19.5	1.18	141	51	52	23	19	1.19	418	60	50
BU60-7-2	142.5	51.5	52.5	23.5	19.5	1.18	142	51.5	52.5	23.5	19.5	1.17	421	60	50
BU60-7-3	142	51	52	23	19	1.18	141.5	52	53	23.5	19.5	1.17	420	60	50
BU110-3-1	142.5	52.5	53.5	24	20	1.15	142	52	53	23.5	19.5	1.15	419	110	100
BU110-3-2	142	51.5	52.5	23.5	19.5	1.16	142	52	53	23.5	19.5	1.16	415	110	100
BU110-3-3	142	51	52	23	19	1.18	143	53	54	24	20	1.17	416	110	100
BU60-3-1	142.5	52.5	53.5	24	20	1.17	142	51.5	52.5	23.5	19.5	1.16	416	60	150
BU60-3-2	143	50	51	22.5	18.5	1.17	143	52	53	23.5	19.5	1.18	418	60	150
BU60-3-3	142	49	50	22	18	1.17	141	50	51	22.5	18.5	1.17	419	60	150

*All dimensions are in millimeters.

Once the specimens were fully constructed, endplates, measuring 360 mm in length, 280 mm in width, and 15 mm in thickness, were welded to both ends of each column. This was done to ensure that the axial compressive force was applied uniformly during testing. Figure 3 illustrates the geometric dimensions of the I-sections used in the experimental program.

The I-sections were created by joining two identical lipped channel sections in a back-to-back configuration, which were then secured with two rows of ST4.8 self-drilling screws, it had a nominal radius of 2.5 mm and a length of 25 mm.

### Material properties

**Fig. 2:**
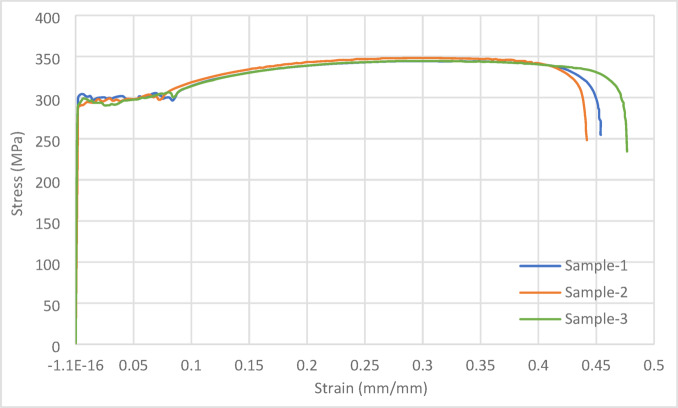
Stress–strain curves obtained from tensile coupon tests on cold-formed steel ^42^.

The material properties of the built-up column specimens were determined through tensile coupon tests, as conducted by Sang L. et al^42^, in accordance with the Chinese Standard GB/T 228.1-2010^43^. To obtain accurate measurements, three longitudinal coupons were cut and milled from the same batch of column specimens. The average values from the coupon tests were as follows: the yield stress (fy) was 292.95 MPa, the tensile strength (fu) was 345.53 MPa, the Young’s modulus (E) was 193.9 GPa, and the elongation at fracture was 45.33% (see Fig. 2). These measured values were used as the material properties for the specimens.

### Dimensions

**Fig. 3:**
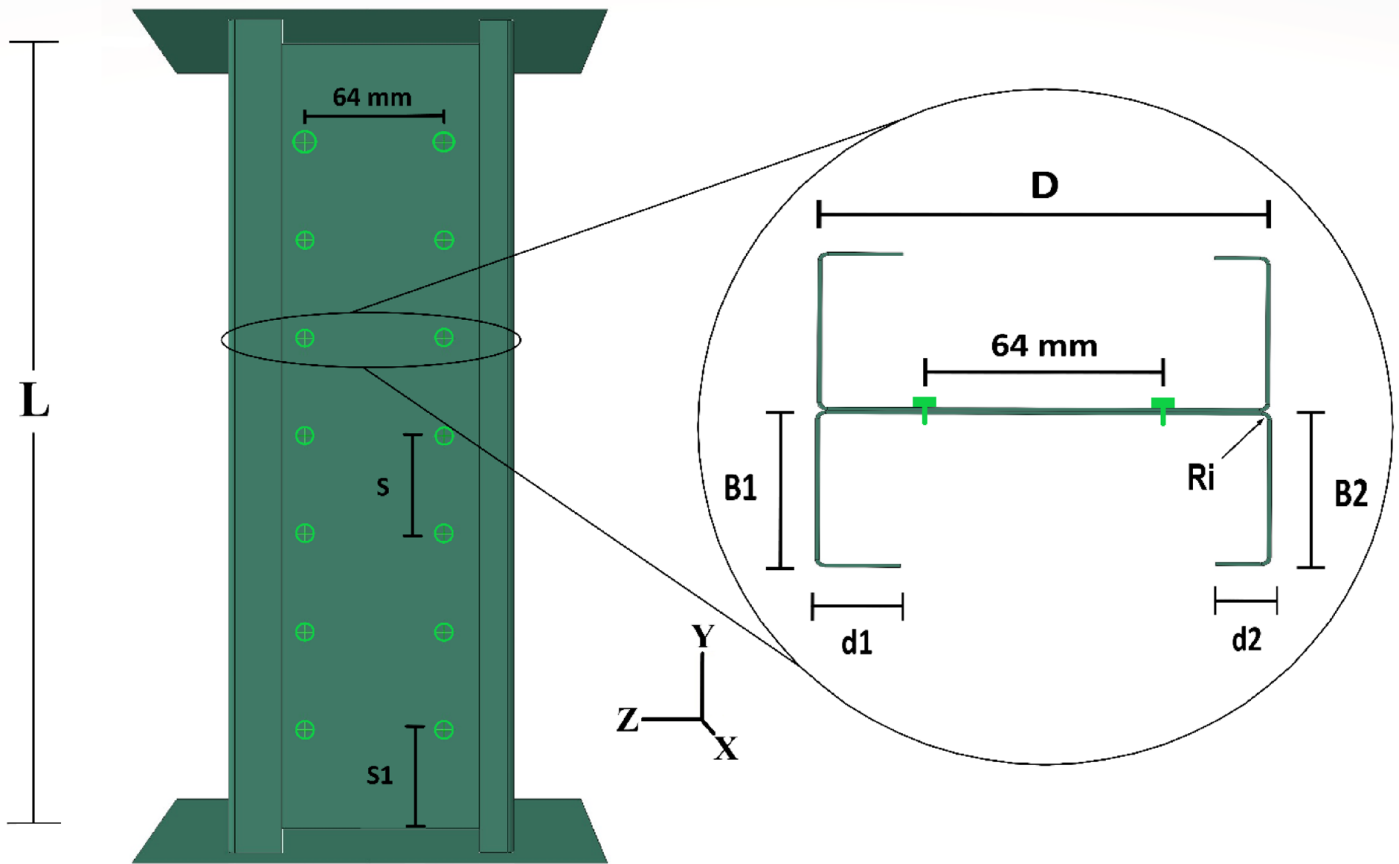
Screw spacing and cross-section dimension symbols for tested specimens.

The constructed columns were designed with two distinct web depths (D), approximately 122 mm and 142 mm. The length of the test specimens was determined as three times the web height, resulting in two different lengths for the built-up columns: approximately 360 mm and 420 mm. The typical flange length (B) was about 52 mm. The average lip length (d) of the cold-formed steel channels was approximately 5% of the column length, leading to two distinct lip lengths: 22 mm for the longer column and 17 mm for the shorter column. Additionally, the internal corner radius (Ri) was 1.5 mm. For further clarification, Fig. 3 provides the symbols, while Table 1 presents the precise measurements of the back-to-back built-up short channel CFS members.

### Screw arrangement

The constructed I-sections were fabricated by joining two identical lipped channel cold-formed steel sections in a back-to-back configuration, secured at the web with two rows of ST4.8 self-drilling screws. These screws were spaced 64 mm apart between the rows. The columns were categorized into two distinct lengths, each subdivided into three groups based on screw spacing. For the shorter columns (L = 420 mm), the screw spacings were 50 mm, 100 mm, and 150 mm. For the smaller columns (L = 360 mm), the screw spacings were 45 mm, 90 mm, and 150 mm. Each column was tested three times, incorporating significant variations in cross-sectional dimensions, as shown in Table 1. Consequently, each main group was further divided into three sub-groups, ensuring comprehensive testing across different configurations.

### Specimen labelling

As previously discussed, the columns were categorized into two main groups based on their lengths: short columns (L = 420 mm) and shorter columns (L = 360 mm). Each group was further subdivided into three classifications according to the screw spacing. To ensure clear identification, the specimens were labeled based on several factors, including the distance from each endplate to the nearest screws (both the first and last screws), as well as the number of screws used to join the back-to-back CFS channel sections.

The labels for the specimens in the first column group (L = 420 mm) include BU60-7, BU110-3, and BU60-3, while those in the second group (L = 360 mm) are labeled BU45-7, BU90-3, and BU105-2.

In the designation “BU60-7-3,” the prefix “BU” denotes the built-up I-section, the number “60” represents the distance from the endplate to the nearest screw, the numeral “7” indicates that seven screws were used in each row to connect the back-to-back channels, and the final numeral “3” corresponds to the third repeated specimen in the series.

### Initial geometrical imperfections

**Fig. 4:**
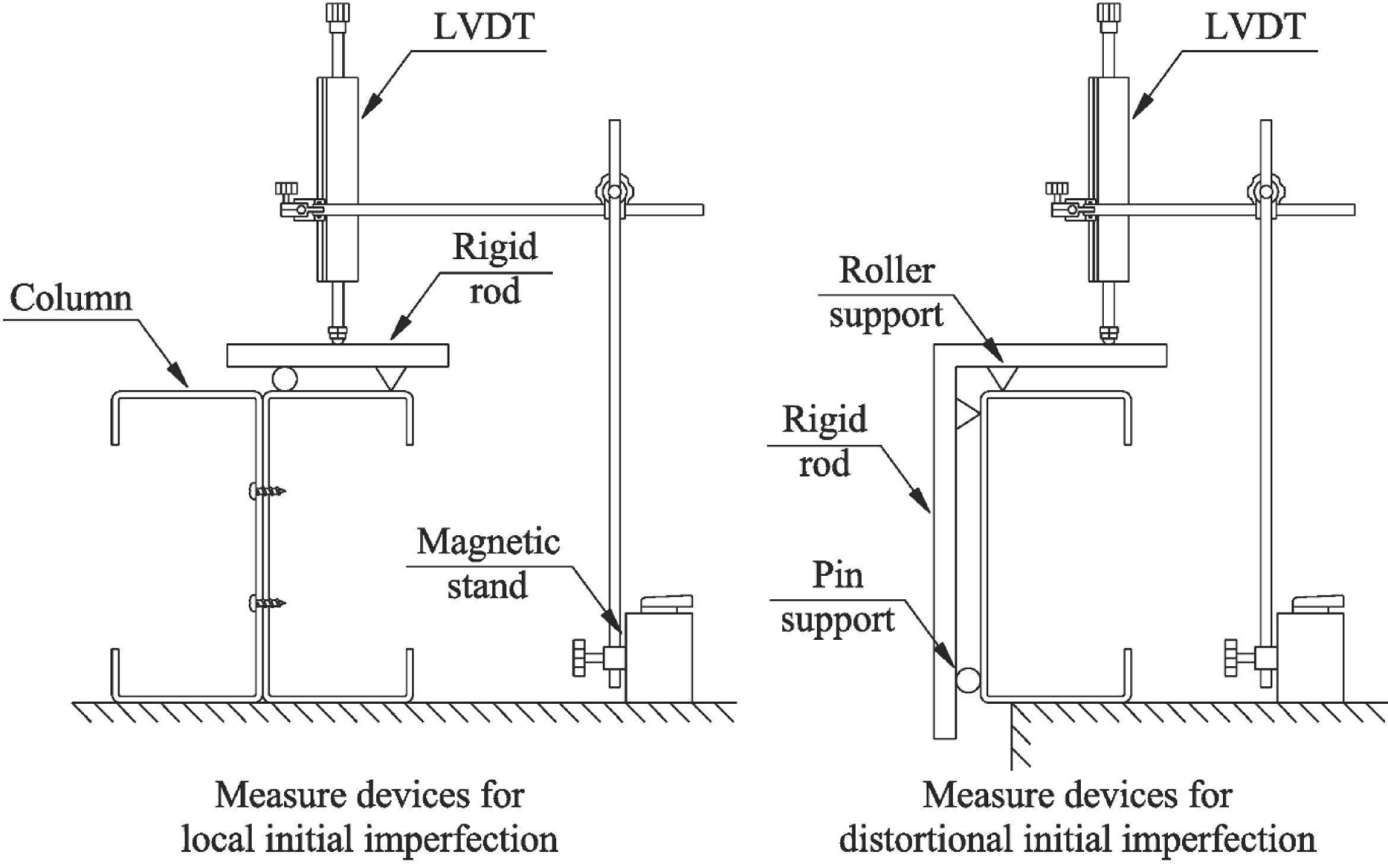
Measurement setup for geometric imperfections.

It is well established that the deformation and axial strength behavior of CFS members are significantly influenced by initial geometric imperfections (*δ)*. Therefore, a systematic quantification of these imperfections is essential prior to testing, as detailed in Table 2. Following the methodology employed by Sang L. et al^42^, a linear variable displacement transducer (LVDT) with an accuracy of 0.001 mm was utilized to measure (*δ)* present in the assembled built-up I-section columns. See Fig. 4.

**Table 2:** The initial imperfection measurement of test specimens and the comparison of experimental tests ^42^ with finite element tests and the direct strength method.

Specimen	Initial Imperfection		P_EXP_	P_FEM_	P_AS/NZS_	P_AS/NZS_/ P_EXP_	P_FEM_ / P_EXP_
	Local (δ_L_)	Distortional (δ_D_)					
	mm		kN			–	
BU45-7-1	0.29	2.46	124	126	114	0.92	1.02
BU45-7-2	0.17	3	123	123	111	0.90	1.00
BU45-7-3	0.21	3.38	121	121	109	0.90	1.00
BU90-3-1	0.22	3.38	119	120	104	0.88	1.01
BU90-3-2	0.17	4.13	119	119	104	0.87	1.00
BU90-3-3	0.27	2.73	118	122	104	0.88	1.03
BU105-2-1	0.25	3.3	117	117	100	0.85	1.00
BU105-2-2	0.21	3.15	117	117	100	0.85	0.99
BU105-2-3	0.23	2.03	117	117	101	0.86	1.00
BU60-7-1	0.23	2	123	125	112	0.91	1.01
BU60-7-2	0.17	3.93	122	122	110	0.90	1.00
BU60-7-3	0.13	2.38	121	124	111	0.91	1.02
BU110-3-1	0.22	2.75	117	118	99	0.84	1.00
BU110-3-2	0.11	1.01	118	121	103	0.88	1.02
BU110-3-3	0.24	2.85	119	118	102	0.86	0.99
BU60-3-1	0.21	1.88	116	117	99	0.85	1.01
BU60-3-2	0.26	2.5	117	120	99	0.85	1.03
BU60-3-3	0.18	3.38	116	116	97	0.84	1.00
Average						0.875	1.008
S						0.026	0.013

A suitable number of strain gauges and linear variable differential transformers (LVDTs) were installed on each specimen to measure the strain and displacement of the column during loading. To accurately determine the critical load associated with local buckling, strain gauges were positioned at the mid-height of the column as well as at locations 100 mm above and below the midpoint. Displacement transducers were placed at the mid-span of each specimen to record the local buckling deformations.

Given that the short columns under consideration are not susceptible to overall buckling, the measurement efforts focused specifically on cross-sectional initial imperfections. The average values of the local initial imperfections (*δ*_L_) and the distortional initial imperfections (*δ*_D_) are presented in Table 2. To clarify the sign convention used, positive values for initial geometrical imperfections indicate an inward rotation of the flange at the flange-web junction or a concave-inward curvature of the plate. Conversely, negative values signify an outward rotation of the flange at the flange-web junction or a concave-outward curvature of the plate.

## Finite element modelling

### General

The finite element method (FEM) and the finite strip method (FSM) are two distinct analytical approaches that can be utilized to analyze thin-walled structures. In this study, the test results were verified using Abaqus 2024 software^44^. The following sub-sections offer a detailed explanation of the key stages in the finite element analysis (FEA), including the model creation, application of boundary conditions, consideration of initial imperfections, and selection of element types and mesh sizes.

### Element type

In the finite element analysis (FEA), the thin-walled member was modeled using S4R shell elements. Specifically, the S4R element available within Abaqus is a four-node quadrilateral shell element characterized by its large-strain capability and reduced integration. Consequently, its computational efficiency and robustness in handling nonlinear problems make it a widely adopted choice for simulating the behavior of thin to moderately thick shell structures.

### Mesh size

**Fig. 5:**
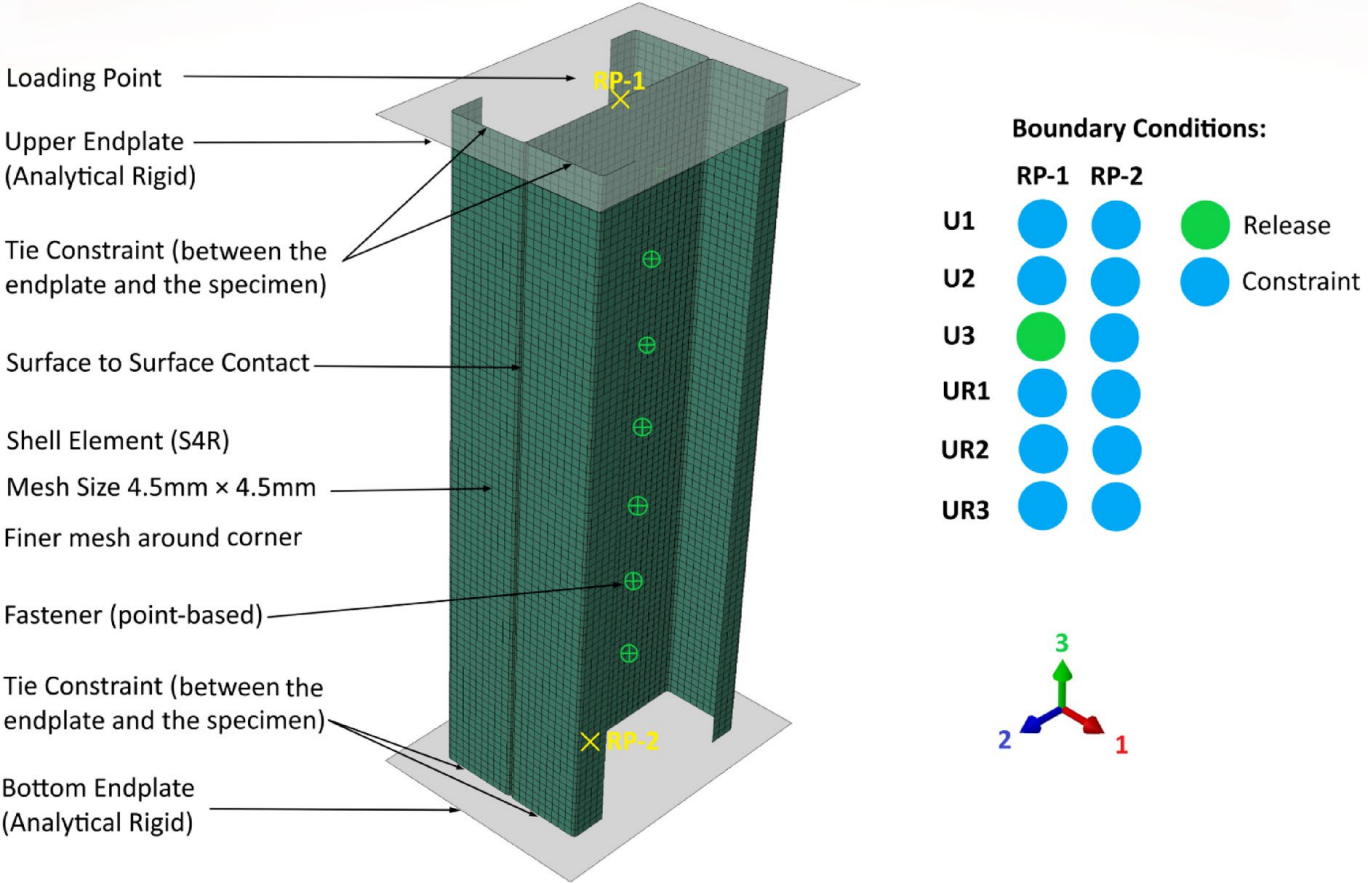
Representative finite element model of built-up back-to-back CFS channel sections.

The accuracy of the FEA results was significantly influenced by the element size. Through a series of trial calculations in the finite element simulation, it was determined that a mesh size of 4.5× 4.5 mm provided results that closely matched the real values, as shown in Fig. 5. Specifically, the mesh size between the junctions of the lips and flanges, as well as between the flanges and the web, was approximately 2.1× 4.5 mm. Additionally, a finer mesh size was applied around the holes and hole stiffeners to capture more precise details in these critical areas.

### Contact between channels

In the finite element model, surface-to-surface contact with finite sliding formulation is applied to represent the interaction between the webs of CFS channels. Two types of contact properties are considered: tangential behavior and normal behavior.

Regarding the tangential behavior, Abaqus defines the interaction between the contacting surfaces in terms of how they slide relative to each other. When the “Frictionless” option is chosen, it assumes that there is no resistance to relative motion in the tangential direction between the surfaces.

In terms of normal behavior, a “hard contact” model is used, which defines pressure-overclosure characteristics. Additionally, the option “Allow separation after contact” is activated, ensuring that once the contact pressure reaches zero, the surfaces can separate freely without the application of tensile forces or adhesion. This approach is particularly suitable for simulations where the contact is temporary, such as in the analysis of cold-formed steel buckling.

### Fastener creation

To model the screw connections between the channels, a two-step process was employed. First, attachment points offset from the edges of the channel webs were defined to establish the precise location of each screw. Subsequently, “Point-based” fasteners were introduced at these predefined points to represent the screws connecting the channels. Each screw was assigned a physical radius of 2.4 mm. Furthermore, to accurately simulate the mechanical behavior of the screws, three translational degrees of freedom (U1, U2, and U3) were activated at the node corresponding to the screw connection point. This allowed the model to capture the potential for translational movement of the screw in all three translational degrees of freedom.

### Boundary conditions

In this study, analytical rigidity was used to model both endplates. A tie constraint was applied between the endplates and the specimens to ensure proper interaction. The reference points RP1 and RP2 were used to establish rigid body constraints with the endplates on either side, thereby facilitating the application of boundary conditions and loading. To simulate fixed boundary conditions, the upper endplate was constrained in five degrees of freedom (three rotational and two translational), while the lower endplate was constrained in six degrees of freedom (three rotational and three translational), as illustrated in Fig. 5. Subsequently, the FEM was subjected to displacement control, with a displacement applied in the 3-direction (−2.8 mm) at reference point RP1.

### Step manager

The selection of appropriate steps plays a critical role in determining the accuracy of the results, particularly for stress–strain curves. In this study, a static general analysis procedure was employed, incorporating an artificial damping factor of 10⁻⁶. Furthermore, the maximum number of increments, as well as the initial, minimum, and maximum increment sizes, were set to 10^6^, 10⁻⁶, 10⁻^55^, and 0.01, respectively, to ensure precise geometrically and materially nonlinear analysis.

For the linear buckling analysis, a “linear perturbation” procedure was used, along with the “Buckle” procedure type. The number of eigenvalues requested, the vectors used per iteration, and the maximum number of iterations were set to 200, 208, and 1000, respectively, to achieve optimal convergence in the analysis.

## Comparison between numerical analysis and experimental results

Due to the small thickness of CFS sections, initial geometric imperfections have a significant impact on their structural behavior. To achieve accurate and precise results, these imperfections must be incorporated into finite element models. Table 2 presents the initial geometric imperfections measured by Sang L et al. ^42^ from experimental tests, which were subsequently integrated into the numerical models.

A comparison is conducted between the results obtained from FEA, experimental tests, and the design standards of Australia and New Zealand (AS/NZS). In Table 2, P_EXP_ represents the axial load capacity of experimental specimens, P_FEM_ denotes the geometrically and materially nonlinear axial load capacity obtained from FEM simulations, and P_AS/NZS_ signifies the linear axial load capacity of specimens based on Australian and New Zealand standards.

The finite element model (FEM) was validated against experimental data from eighteen CFS channel short columns with varying cross-sectional dimensions and screw spacing. The modeling incorporated the measured dimensions and material properties of the tested columns. A comprehensive comparison was conducted, analyzing ultimate strengths, axial load vs. axial shortening curves, and deformed shapes of the columns.

A comparison of ultimate strengths obtained from experimental tests (P_EXP_), finite element analysis (P_FEA_), and the Australian/New Zealand standards (P_AS/NZS_) is presented in Table 2. The mean ultimate load capacity ratio of FEA to EXP is 1.008, with a sample standard deviation (S) of 0.013. The small standard deviation indicates that the data points are closely clustered around the mean, confirming consistent and reliable findings. This enhances structural stability and ensures that FEM accurately reflects experimental behavior. The close agreement between FEA and experimental results is likely due to precise measurements of dimensions, material properties, and initial geometric imperfections of the built-up columns.

Conversely, the mean ultimate load capacity ratio of AS/NZS to EXP is 0.875, with a sample standard deviation (S) of 0.026. This result indicates that the ultimate bearing capacities derived from DSM formulas in AS/NZS are underestimated by approximately 12.5% compared to experimental results. While, Chi Y et al^28^ determined that the AS/NZS & AISI exhibit a conservatism of approximately 9% relative to the experimental results for plain channel sections. On the other hand, Roy K. et al^45^ demonstrated that the AS/NZS are not conservative for stub and short columns that failed due to local buckling. However, Yao X^9^ demonstrates that CFS built-up columns that fail in distortional buckling and interactive buckling are not safe for the existing DSM. This discrepancy is likely due to the AS/NZS reliance on yield stress without incorporating ultimate strength, leading to conservative capacity predictions.

**Fig. 6:**
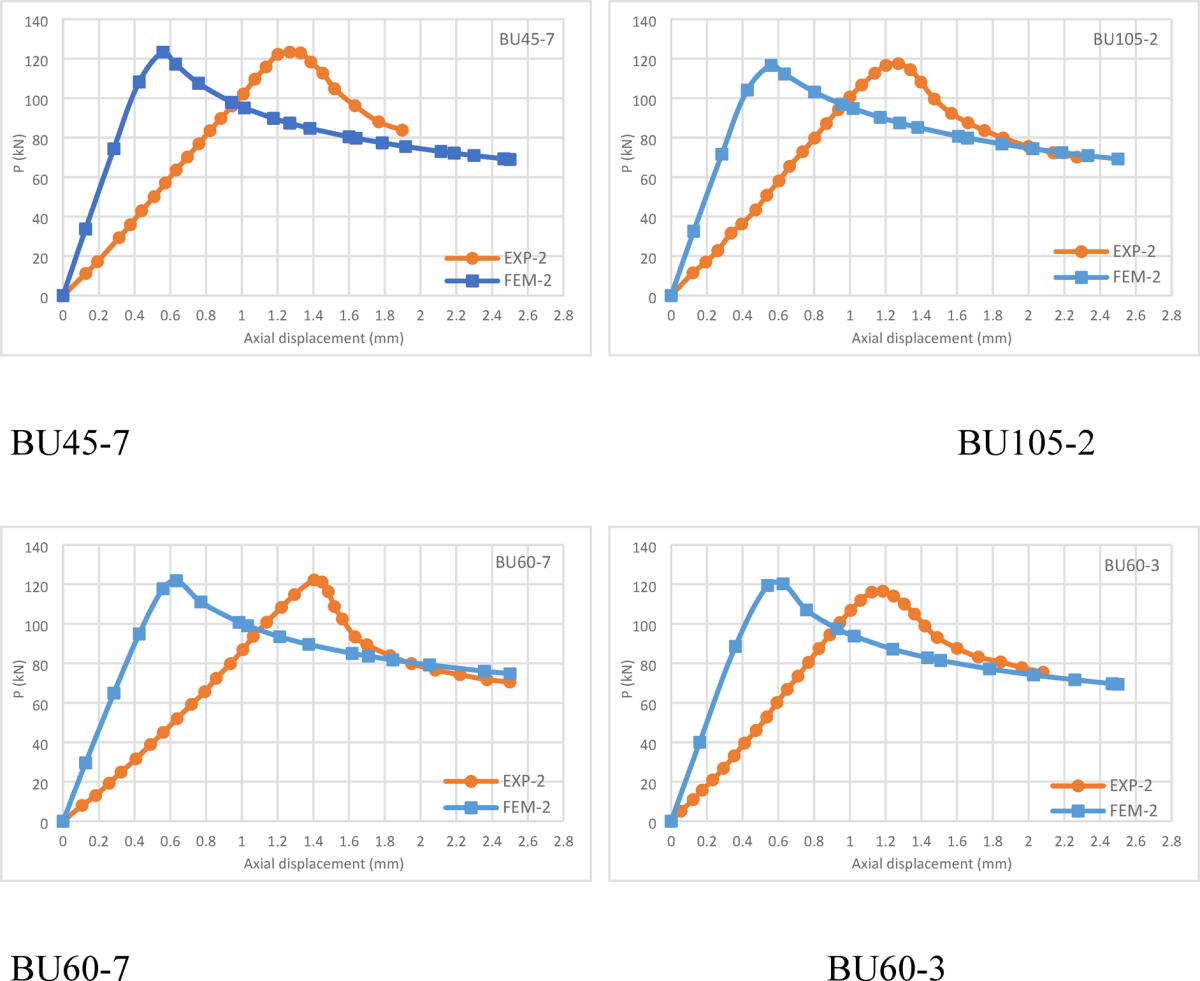
Load-axial displacement curve for experimental ^42^ and FEM model specimens.

The axial load versus axial shortening curves obtained from FEA and experimental testing are compared in Fig. 6. A strong correlation is observed between the experimental and numerical results, demonstrating the accuracy of the FEM in predicting structural behavior.

Both the experimental and FEM curves exhibit a linear increase in load capacity until reaching the ultimate strength, followed by a gradual decline of approximately 30% upon failure. However, the observed difference in axial displacement at ultimate strength between the finite element models (approximately 0.6 mm) and the experimental tests (approximately 1.2 mm) can be attributed to several factors. In the FEM models, it is possible to precisely define ideal boundary conditions, such as fixed–fixed ends. However, in experimental tests, minor deviations due to human or setup error may result in boundary conditions that deviate slightly from the intended fixed–fixed configuration.

Additionally, the FEM models assume perfectly rigid screw connections, whereas in reality, some flexibility or deformation may occur at the screw joints during testing. Another contributing factor is the assumption of frictionless contact between the channel sections in the FEM models, which simplifies the simulation but does not reflect the actual contact behavior in physical tests, where some friction is inevitably present.

These differences in modelling assumptions and experimental constraints can lead to slight discrepancies in the displacement at which ultimate strength is reached.

**Fig. 7:**
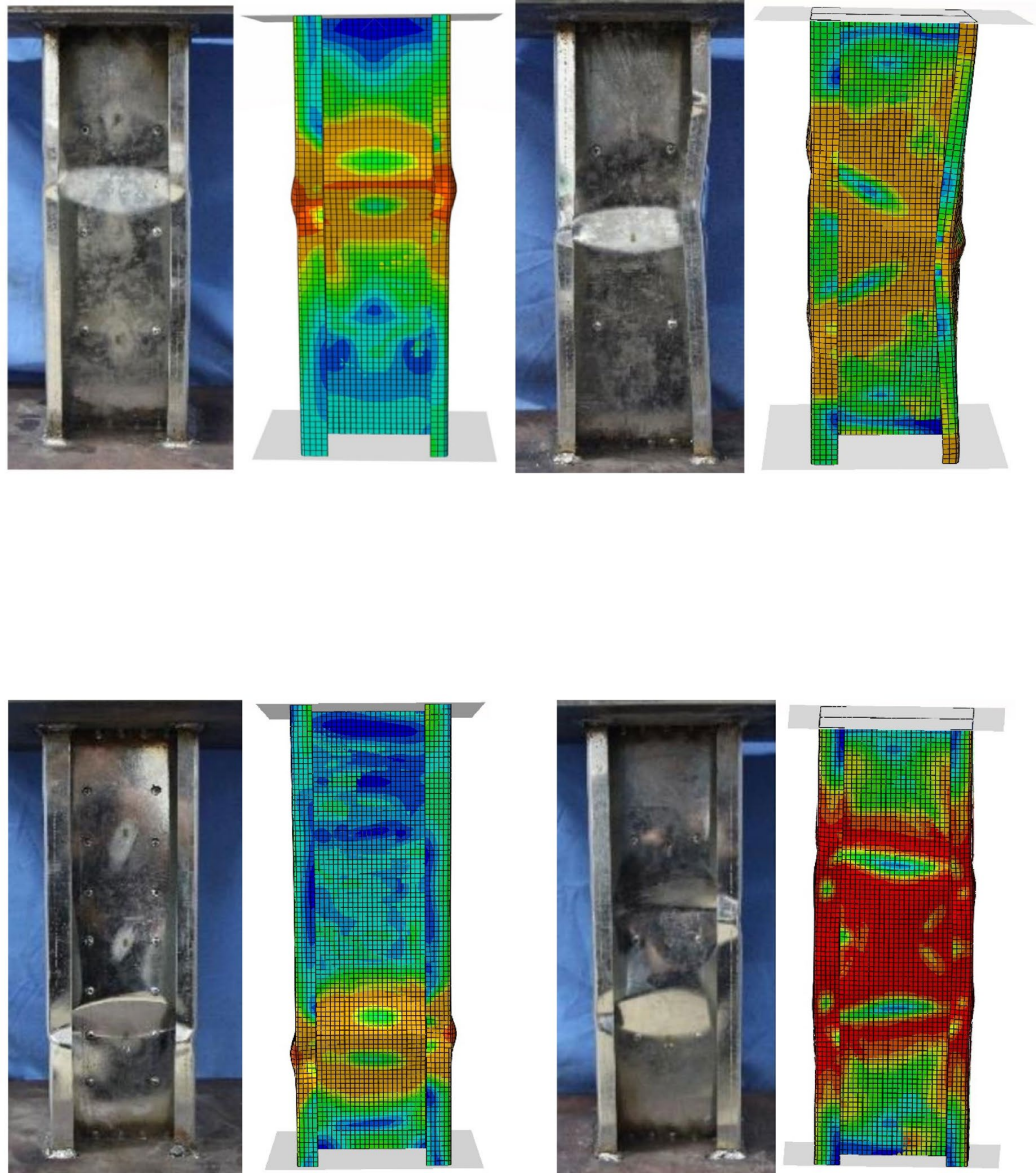
Failure modes of experimental ^42^ and finite element models.

The failure modes observed in experimental tests and finite element models (FEM) are compared in Fig. 7. A strong correlation is observed between the experimental and numerical failure shapes, indicating the reliability of the FEM in capturing structural behavior.

The comparison between experimental and FEA results confirms that the proposed FEM accurately predicts the failure patterns of built-up CFS channel short columns, demonstrating its effectiveness in structural analysis.

## Numerical investigation

The validated finite element models will be utilized for further numerical investigations, focusing on the effect of web holes on built-up I-channel short columns. In this study, four types of holes are introduced at the centroid of the plain webs of built-up I-sections, both with and without edge stiffeners. These hole shapes include circular, square, rectangular, and slotted holes, each tested with and without edge-stiffened reinforcements.

**Fig. 8:**
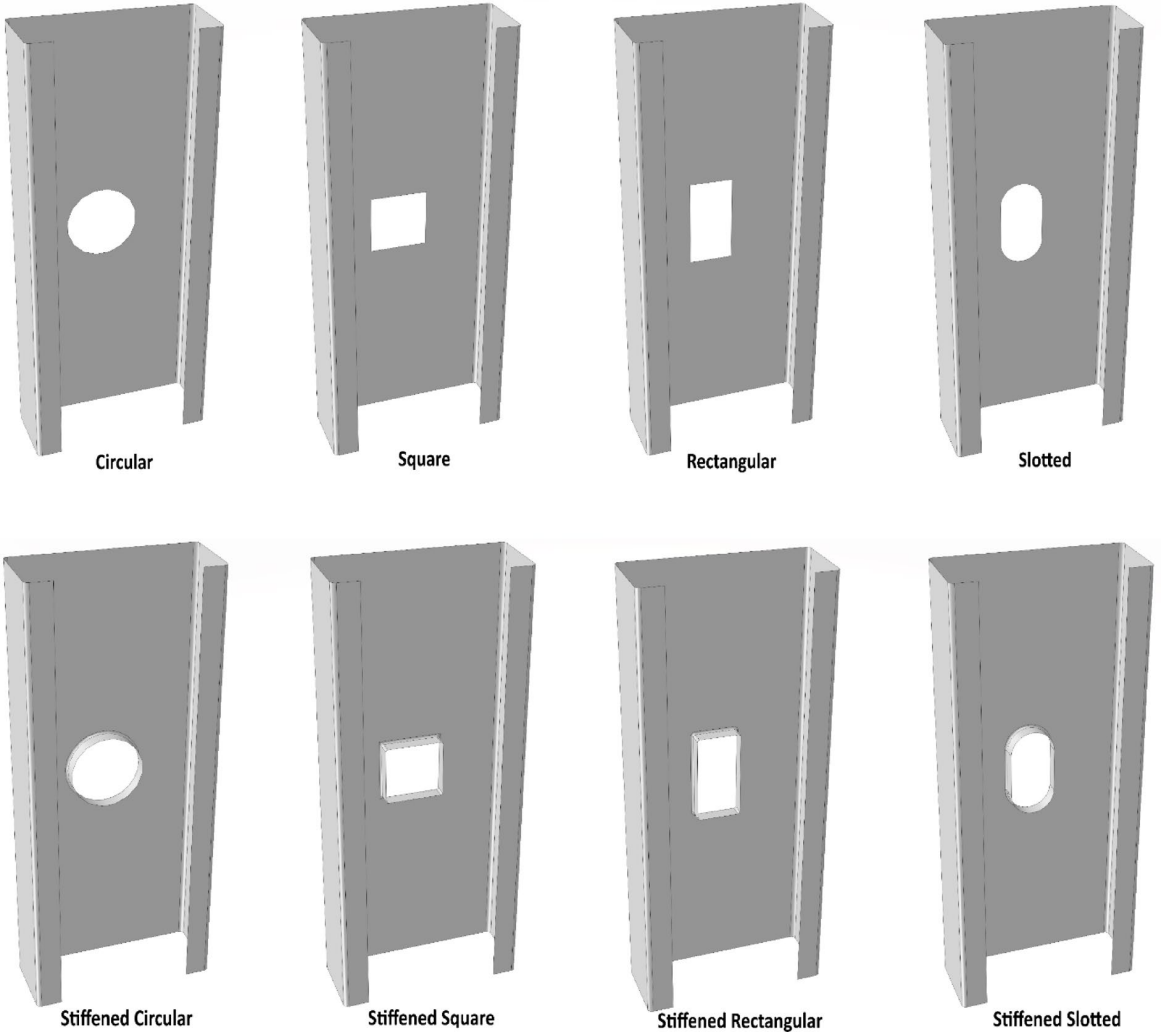
Detailed drawing of unstiffened and edge-stiffened holes in FEM specimens.

To ensure a fair comparison among different hole shapes, the same hole area is maintained across all specimens. Specifically, the area of each hole is set to 1800 mm^2^. Moreover, the edge-stiffener length for all specimens is 10 mm, with an inside bent radius of 1.5 mm between the stiffener and the web. Figure 8 illustrates the various hole configurations, highlighting their structural differences.

It is important to note that all four-hole shapes considered in this study have the same cross-sectional area. Specifically, the area of the circular hole is calculated as πr^2^, where r is the radius equal to the half-width of the equivalent square hole. The square hole has an area equal to the square of its width, with equal width and depth. For the rectangular hole, the depth is twice the width, maintaining the same total area. The slotted hole consists of two half-circular ends connected by a central rectangular portion, forming a composite shape with an area equivalent to the other hole types.

Edge-stiffened and unstiffened web holes are openings that are generated in the webs of CFS section columns. Reinforcements (additional flange or lip) are incorporated around the margins of the web holes, which enhance axial load-carrying capacity^27,28,46,47^, reduce stress concentrations, and improve structural stability. Conversely, unstiffened web openings are devoid of these reinforcements, rendering them more susceptible to local buckling^48^ and diminishing the column’s overall strength^49–51^. The column’s performance under axial loading is substantially influenced by the presence and type of web holes.

In order to facilitate the installation of electrical, sewage, or heating systems, it is frequently necessary to create web holes in the structural elements to reduce their weight^52^.

Previously, the strong agreement between experimental and FEM results was established, with an average difference in axial load capacity of only 0.8% and a low (S) of 0.013. Building upon this validation, the back-to-back plain CFS short channel model was further investigated. Initially, a circular hole with an area of 1800 mm^2^ was introduced at the centroid of the web. Subsequently, while maintaining the same 1800 mm^2^ hole area, an edge stiffener, or lip, with a 10 mm length was added around the perimeter of the circular hole. This modification allowed for the direct comparison of the structural behavior with and without the edge stiffener.

The introduction of un-stiffened circular holes in the webs of built-up CFS I-channel short columns results in a reduction in axial load capacity. Specifically, the ratio of the average axial load capacity of FEMs with un-stiffened circular holes to that of experimental tests with plain webs (P_FEM_ {un-stiffened circular holes}/P_EXP_ {plain webs}) is 0.91, with a (S) of 0.04, as shown in Table 3. This finding indicates that the introduction of holes leads to an approximate 9.35% decrease in the axial load capacity of the columns.

**Table 3:** Comparison of axial load carrying capacity of plain webs ^42^, unstiffened, and edge-stiffened circular holes.

Specimen	plain webs	Un-stiffened holes			Edge-stiffened holes		
	P_EXP_	P_FEM_	P_FEM_/P_EXP_	Strength changes due to holes	P_FEM_	P_FEM_/P_EXP_	Strength changes due to holes
	kN	kN	–	%	kN	–	%
BU45-7-1	124	106	0.86	− 14.0	127	1.02	2.3
BU45-7-2	123	114	0.93	− 7.5	133	1.08	7.6
BU45-7-3	121	110	0.91	− 9.1	130	1.07	7.3
BU90-3-1	119	102	0.86	− 14.1	119	1.00	0.0
BU90-3-2	119	103	0.87	− 13.3	120	1.01	0.5
BU90-3-3	118	111	0.94	− 5.8	128	1.08	8.3
BU105-2-1	117	105	0.90	− 10.2	120	1.02	2.1
BU105-2-2	117	108	0.92	− 8.3	123	1.04	4.4
BU105-2-3	117	101	0.86	− 13.7	117	1.00	-0.4
BU60-7-1	123	119	0.97	− 2.9	121	0.99	−1.1
BU60-7-2	122	119	0.97	− 2.9	119	0.97	−2.5
BU60-7-3	121	106	0.88	− 12.5	118	0.97	−2.5
BU110-3-1	117	113	0.96	− 3.9	113	0.97	−3.3
BU110-3-2	118	102	0.87	− 13.0	113	0.96	−4.0
BU110-3-3	119	105	0.88	− 11.9	116	0.97	−2.6
BU60-3-1	116	104	0.89	− 10.7	111	0.95	−4.6
BU60-3-2	117	111	0.95	− 4.6	117	1.01	0.6
BU60-3-3	116	104	0.90	− 9.9	114	0.99	-0.9
Average			0.91	− 9.35		1.01	0.61
S			0.04	0.04		0.04	0.04

On the other hand, incorporating edge-stiffened circular holes has a positive effect on structural performance. The ratio of the average axial load capacity of finite element models with edge-stiffened circular holes to that of experimental tests with plain webs (P_FEM_ {edge stiffened circular holes}/P_EXP_ {plain webs}) is 1.01, with a (S) of 0.04, see Table 3. This result suggests that, despite the presence of holes, the axial load capacity increases by approximately 0.61%. The addition of stiffeners around the web holes enhances the structural integrity of the column, compensating for the strength reduction caused by the perforations.

A comparative analysis was conducted between experimental plain web specimens, finite element models (FEM) with un-stiffened square holes, and FEM with edge-stiffened square holes. The average axial load capacity of FEM with un-stiffened square holes relative to experimental tests with plain webs (P_FEM_ {un-stiffened square holes}/P_EXP_ {plain webs}) was found to be 0.92, with a (S) of 0.04, see Table 4. This result indicates that introducing un-stiffened square holes in the webs leads to an approximate 8.43% reduction in the axial load capacity of the built-up CFS I-channel short columns.

**Table 4:** Comparison of axial load carrying capacity for plain webs ^42^, unstiffened, and edge-stiffened square hole configurations.

Specimen	plain webs	Un-stiffened holes			Edge-stiffened holes		
	P_EXP_	P_FEM_	P_FEM_/P_EXP_	Strength changes due to holes	P_FEM_	P_FEM_/P_EXP_	Strength changes due to holes
	kN	kN	–	%	kN	–	%
BU45-7-1	124	106	0.86	− 14.0	126	1.02	2.1
BU45-7-2	123	113	0.92	− 8.0	132	1.07	7.3
BU45-7-3	121	110	0.91	− 9.0	130	1.07	7.0
BU90-3-1	119	102	0.86	− 13.8	118	0.99	− 0.9
BU90-3-2	119	104	0.87	− 12.8	119	1.00	− 0.1
BU90-3-3	118	112	0.95	− 4.9	127	1.08	7.9
BU105-2-1	117	105	0.90	− 10.2	121	1.04	3.5
BU105-2-2	117	108	0.92	− 7.9	120	1.03	2.5
BU105-2-3	117	106	0.91	− 9.3	111	0.95	− 5.4
BU60-7-1	123	106	0.87	− 13.3	122	0.99	− 0.7
BU60-7-2	122	119	0.98	− 2.3	119	0.97	− 2.8
BU60-7-3	121	120	0.99	− 1.4	118	0.97	− 2.7
BU110-3-1	117	110	0.93	− 6.6	113	0.96	− 3.6
BU110-3-2	118	101	0.86	− 14.5	114	0.97	− 3.5
BU110-3-3	119	109	0.92	− 8.4	116	0.97	− 2.7
BU60-3-1	116	113	0.97	− 3.0	111	0.95	− 4.6
BU60-3-2	117	113	0.97	− 3.4	118	1.01	1.5
BU60-3-3	116	105	0.91	− 9.3	114	0.99	− 1.1
Average			0.92	− 8.43		1.00	0.21
S			0.04	0.04		0.04	0.04

On the other hand, when edge stiffeners were added around the square holes, the average axial load capacity of FEM with edge-stiffened square holes relative to experimental tests with plain webs (P_FEM_ {edge-stiffened square holes}/P_EXP_ {plain webs}) increased to 1.00, with a (S) of 0.04, as shown in Table 4. This finding suggests that, despite the presence of square holes, the axial load capacity slightly increased by 0.21%. The improvement in strength is attributed to the reinforcing effect of the stiffener around the web holes, which helps mitigate the reduction in load-bearing capacity caused by the openings.

Concerning the comparison between experimental plain web specimens and FEM models incorporating rectangular holes, both unstiffened and edge-stiffened, it is important to recall that all holes maintain a consistent area of 1800 mm^2^, with the length of the rectangular holes being twice their width.

Initially, the ratio of the average axial load capacity of specimens with unstiffened rectangular holes to that of plain web specimens (P_FEM_ {unstiffened rectangular holes}/P_EXP_ {plain webs}) was found to be 0.92, with a (S) of 0.04, as detailed in Table 5. This result indicates a decrease of approximately 7.83% in the axial load capacity of the built-up CFS I-channel short columns due to the introduction of the unstiffened rectangular holes.

**Table 5:** Evaluation of axial load bearing capacity of plain webs ^42^, unstiffened, and edge-stiffened rectangular openings.

Specimen	plain webs	Un-stiffened holes			Edge-stiffened holes		
	P_EXP_	P_FEM_	P_FEM_/P_EXP_	Strength changes due to holes	P_FEM_	P_FEM_/P_EXP_	Strength changes due to holes
	kN	kN	–	%	kN	–	%
BU45-7-1	124	107	0.87	− 13.2	125	1.01	0.9
BU45-7-2	123	115	0.93	− 7.1	125	1.02	1.6
BU45-7-3	121	112	0.92	− 8.0	123	1.02	1.9
BU90-3-1	119	104	0.88	− 12.5	118	0.99	− 0.9
BU90-3-2	119	105	0.88	− 11.8	117	0.98	− 1.6
BU90-3-3	118	118	1.00	0.1	127	1.07	7.2
BU105-2-1	117	108	0.92	− 7.8	119	1.01	1.4
BU105-2-2	117	110	0.94	− 6.5	120	1.02	2.4
BU105-2-3	117	104	0.88	− 11.6	119	1.02	1.8
BU60-7-1	123	108	0.88	− 11.8	120	0.98	− 2.4
BU60-7-2	122	108	0.89	− 11.3	120	0.98	− 2.0
BU60-7-3	121	108	0.89	− 10.9	121	1.00	0.0
BU110-3-1	117	112	0.95	− 4.5	112	0.96	− 4.4
BU110-3-2	118	106	0.90	− 10.0	111	0.94	− 5.6
BU110-3-3	119	116	0.98	− 2.2	117	0.98	− 1.8
BU60-3-1	116	114	0.98	− 1.7	116	0.99	− 0.5
BU60-3-2	117	114	0.98	− 1.8	114	0.98	− 1.8
BU60-3-3	116	106	0.92	− 8.5	116	1.00	0.0
Average			0.92	− 7.83		1.00	− 0.21
S			0.04	0.04		0.03	0.03

Subsequently, the ratio of the average axial load capacity of specimens with edge-stiffened rectangular holes to that of plain web specimens (P_FEM_ {edge-stiffened rectangular holes}/P_EXP_ {plain webs}) was calculated to be 1.00, with a (S) of 0.03, also presented in Table 5. This latter finding reveals that, despite the presence of the rectangular holes, the addition of edge stiffeners resulted in only a minimal decrease of approximately 0.21% in the average axial load capacity. Consequently, the observed improvement in strength can be attributed to the stiffening effect provided by the edge stiffeners around the web holes.

Turning now to the comparison between experimental plain web specimens and their corresponding Finite Element Models (FEMs) with slotted holes, both with and without edge stiffening, several key observations can be made. Slotted holes consist of a square hole combined with two halves of circular holes, and as previously mentioned, each hole has a total area of 1800 mm^2^.

Firstly, the ratio of the average axial load capacity of specimens with unstiffened slotted holes to that of plain web specimens (P_FEM_ {unstiffened slotted holes}/P_EXP_ {plain webs}) is 0.92, with a (S) of 0.03, as shown in Table 6. This result indicates that the introduction of unstiffened slotted holes leads to a reduction of approximately 8.22% in the axial load capacity of built-up CFS I-channel short columns.

**Table 6:** Axial load carrying capacity comparison of plain webs ^42^, unstiffened slotted webs, and edge-stiffened slotted webs.

Specimen	plain webs	Un-stiffened holes			Edge-stiffened holes		
	P_EXP_	P_FEM_	P_FEM_/P_EXP_	Strength changes due to holes	P_FEM_	P_FEM_/P_EXP_	Strength changes due to holes
	kN	kN	–	%	kN	–	%
BU45-7-1	124	108	0.87	− 12.7	119	0.96	− 3.7
BU45-7-2	123	115	0.93	− 6.6	131	1.07	6.5
BU45-7-3	121	112	0.92	− 7.9	124	1.02	2.4
BU90-3-1	119	105	0.88	− 11.7	118	0.99	− 0.6
BU90-3-2	119	106	0.89	− 11.5	118	0.99	− 0.8
BU90-3-3	118	114	0.96	− 3.8	127	1.08	7.5
BU105-2-1	117	109	0.93	− 7.4	119	1.01	1.5
BU105-2-2	117	111	0.94	− 5.9	120	1.03	2.5
BU105-2-3	117	105	0.89	− 10.9	115	0.98	− 2.4
BU60-7-1	123	109	0.89	− 11.3	123	1.00	0.4
BU60-7-2	122	118	0.96	− 3.7	117	0.96	− 3.9
BU60-7-3	121	108	0.89	− 11.3	117	0.96	− 3.6
BU110-3-1	117	107	0.91	− 9.1	112	0.95	− 4.7
BU110-3-2	118	106	0.90	− 9.8	117	0.99	− 0.6
BU110-3-3	119	111	0.93	− 6.8	116	0.98	− 2.0
BU60-3-1	116	108	0.92	− 7.6	116	0.99	− 0.7
BU60-3-2	117	114	0.98	− 1.8	117	1.01	0.6
BU60-3-3	116	106	0.92	− 8.2	115	1.00	− 0.1
Average			0.92	− 8.22		1.00	− 0.10
S			0.03	0.03		0.03	0.03

Secondly, when edge stiffeners are incorporated around the slotted holes, the ratio of average axial load capacity (P_FEM_ {edge-stiffened slotted holes}/P_EXP_ {plain webs}) increases to 1.00, with a (S) of 0.03, as presented in Table 6. This demonstrates that, despite the presence of slotted holes, the addition of edge stiffeners effectively mitigates the reduction in strength. In fact, the decrease in axial load capacity is reduced to a negligible 0.10%, highlighting the positive impact of stiffeners in maintaining the structural integrity of the columns.

**Fig. 9:**
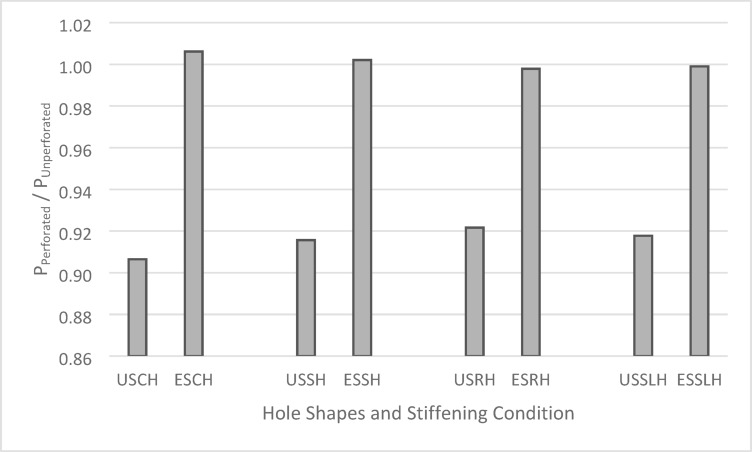
Comparison of Axial Strength Ratios Based on Hole Shapes and Stiffening.

In summary, the presented results demonstrate the significant influence of web holes and edge stiffeners on the axial load-carrying capacity of built-up back-to-back CFS channel short columns. While the shape of the web holes appears to have a comparatively minor impact on the axial load capacity, the presence of the holes and the application of edge stiffeners are crucial factors. Figure 9 presents the axial‐strength ratios of perforated CFS I‐shaped columns, both stiffened and unstiffened, normalized by the strength of their unperforated counterparts, together with a comparison across various hole geometries. The abbreviations used in Fig. 9 are defined as follows: USCH, unstiffened circular hole; ESCH, edge-stiffened circular hole; USSH, unstiffened square hole; ESSH, edge‐stiffened square hole; USRH, unstiffened rectangular hole; ESRH, edge‐stiffened rectangular hole; USSLH, unstiffened slotted hole; and ESSLH, edge‐stiffened slotted hole.

Specifically, introducing 1800 mm^2^ holes into the webs, regardless of their shape, leads to an approximate 8.46% reduction in axial load capacity when no edge stiffening is employed. However, by incorporating 10 mm stiffeners around the perimeter of these same 1800 mm^2^ holes, the detrimental effect of the holes is mitigated.

Indeed, with the addition of stiffeners, the axial load capacity of the validated specimens actually increases by approximately 0.13%, despite the continued presence of the holes. This clearly highlights the effectiveness of edge stiffening in optimizing, and even slightly enhancing, the load-bearing capacity of these structural members.

## Conclusion

This study presents a numerical investigation into the axial strength behavior of 180 built-up back-to-back cold-formed steel (CFS) short lipped channel columns. The finite element model (FEM) was initially validated against experimental data from eighteen CFS short columns reported in the literature. A strong agreement between the geometrically and materially nonlinear finite element predictions and experimental test results was observed, with a mean ultimate load capacity ratio (FEM/EXP) of 1.008 and a standard deviation of 0.013. Additionally, the study highlighted that the Direct Strength Method (DSM) predictions within the Australian/New Zealand Standards (AS/NZS) conservatively underestimate the axial load capacity by approximately 12.5%, as evidenced by a mean load capacity ratio (AS/NZS/EXP) of 0.875 and a standard deviation of 0.026.

Upon validation, the verified FEM was employed to examine the influence of various web hole shapes, with and without edge stiffeners, on the axial load capacity of built-up I-shaped columns. All web openings considered had a uniform area of 1800 mm^2^, and the perimeter stiffeners, where employed, had a width of 10 mm. A comparison of the perforated FEM models with experimental results for plain web columns yielded several key findings. The introduction of unstiffened circular holes resulted in an approximate 9.35% reduction in axial load capacity, while edge-stiffened circular holes led to a slight increase of 0.61%. Similarly, unstiffened square holes caused an 8.43% reduction, whereas edge-stiffened square holes restored the capacity, resulting in a negligible 0.21% increase. Unstiffened rectangular holes reduced the axial load capacity by approximately 7.83%, while edge-stiffened rectangular holes exhibited only a minor reduction of 0.21%. Lastly, unstiffened slotted holes caused an 8.22% decrease; however, the incorporation of edge stiffeners effectively mitigated this reduction, limiting the decrease to a minimal 0.10%.

The results clearly demonstrate that the presence of web holes significantly affects the axial load-carrying capacity of built-up CFS I-section short columns. However, the specific shape of the openings has a relatively minor impact on the overall load capacity. More importantly, the incorporation of edge stiffeners around the web perforations proves to be a highly effective strategy for mitigating strength reduction, optimizing structural performance, and even slightly enhancing load-bearing capacity. Based on these findings, the use of edge stiffeners around web openings is strongly recommended to improve the structural integrity and efficiency of perforated built-up CFS columns.

As a recommendation for future research, it would be valuable to investigate the effects of various hole geometries, both with and without edge stiffeners, as well as the influence of different hole areas on the axial load-carrying capacity of cold-formed steel (CFS) built-up sections. Such studies could offer important insights into optimizing the design of perforated CFS structural members for enhanced performance and structural efficiency.

## Supplementary Information

Below is the link to the electronic supplementary material.

Supplementary Material 1

## Data Availability

All data generated or analysed during this study are included in this published article [and its supplementary information files].
